# Supporting evidences for potential biomarkers of endometriosis detected in peripheral blood^☆^

**DOI:** 10.1016/j.dib.2015.10.047

**Published:** 2015-11-17

**Authors:** Pietro G. Signorile, Alfonso Baldi

**Affiliations:** Fondazione Italiana Endometriosi, Rome, Italy

## Abstract

Incidence of endometriosis is very high in women in the reproductive age (around 10%). To date, a reliable non-invasive diagnostic test for early diagnosis of endometriosis is not available. In this article we describe the potential value as diagnostic markers for endometriosis of two proteins (serum albumin and complement C3 precursor), previously identified as differentially expressed in women with endometriosis respect to healthy control by 2D gel analysis. A detailed description of the results obtained with this proteomic approach can be found in Signorile and Baldi [1]. ELISAs were performed on a large cohort of endometriosis (*n*=100) and healthy patients (*n*=10) to establish the differential expression of the identified proteins. ROC analyses confirmed the statistical significance of the differential expression of these proteins: serum albumin (*p*=0.028) ad complement C3 precursor (*p*=0.082). Evaluation of these two proteins, together with the already described Zn-alpha2-glycoprotein [1], could help in the early identification of endometriosis patients

**Specifications Table**

**Table:** 

Subject area	*Medicine*
More specific subject area	*Gynecology*
Type of data	*Table, text file, graph, figure*
How data was acquired	*ELISA tests*
Data format	*Raw, analyzed, and ROC curves*
Experimental factors	*We compared the expression of two proteins in sera from endometriosis patients and from healthy controls*
Experimental features	*ELISA tests were performed using commercially available kits*
Data source location	*Blood samples were collected in Italy by Fondazione Italiana Endometriosi*
Data accessibility	*N/A*

**Value of the data**

• Definition of the expression of serum albumin and C3 complement could help identify patients suffering for endometriosis at an early stage.
• Assessment of the expression of the two proteins in the serum by ELISA make this analysis easily reproducible.
• Definition of the experimental condition to detect these two proteins, together with the already described Zn-alpha2-glycoprotein [1], represents the rational basis for simultaneously analysis of these potential biomarkers in a wide cohort of endometriosis patients.

## 1 Data

**Fig. 1:**
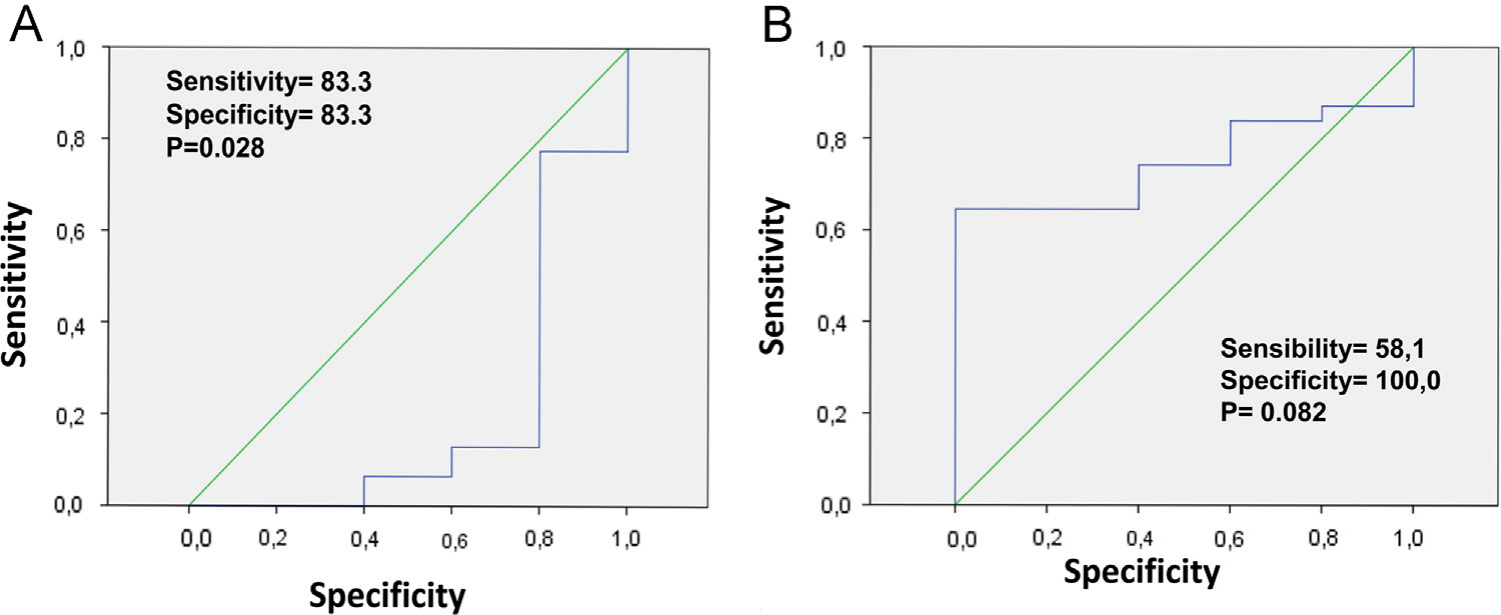
ROC curves for (A) human serum albumin and (B) complement C3, together with specificity and sensitivity.

Endometriosis is one of the most common gynecological diseases in women during the reproductive age [2], [3]. Despite of this, endometriosis is one of the human diseases where a very long time-interval exists between insurgence of the symptoms and final diagnosis, making it one of the most under-diagnosed and under-treated disease [4], [5], [6], [7]. Therefore, the clinical value of a non-invasive diagnostic test for endometriosis would be enormous, because it could allow to immediately identify among women with sub-fertility, those suffering of endometriosis and to rapidly perform laparoscopic surgery, that has been reported to increase fertility [8], [9]. ELISA tests were performed on a cohort of endometriosis (*n*=100) and healthy patients (*n*=10) in order to confirm the differential expression of two proteins, complement C3 and human albumin identified through the proteomic approach described in Signorile and Baldi [1]. Interestingly, most of the albumin was depleted before performing the 2D gel analysis. However, albumin was still identified among the differential expressed markers as it is stated in Table 3 of the original paper [1]. ROC analysis of ELISA results confirmed the statistical significance of the differential expression for these proteins in endometriosis patients respect to healthy controls. In Fig. 1, the ROC curves for these proteins are depicted, together with specificity and sensitivity.

## 2 Experimental design, materials and methods

### 2.1 Sample collection and serum preparation

Details about the recruitment of the human subjects can be found in Signorile and Baldi [1]. Briefly, the control group included 10 healthy women in reproductive age, with no clear signs of endometriosis, such as irregular cycling and ovulating, and that had at least one normal pregnancy. The group of endometriosis patients included only subjects where the diagnosis of endometriosis was definitively confirmed by laparoscopy and histology. Exclusion criteria in the group of endometriosis patients were: a) patients using hormonal medication; b) patients operated within 6 months before the time of sample collections and c) patients suffering of degenerative, autoimmune or neoplastic diseases. The clinical characteristics of the patients enrolled are described in Signorile and Baldi [1]. Analysis was done on a subset of the patient enrolled in the previous paper. A specific selection of the patients was not performed, but all the samples still available for the ELISA assay were used. Ten milliliters of peripheral blood were obtained from patients by venipuncture, collected in EDTA tubes and stored at +4 °C. In order to collect sera, samples were centrifuged at 3000 rpm for 10 min at 4 °C, aliquoted in sterilized tubes, labeled and stored at −80°C. Samples that were haemolysed during the procedure, were not included in the study protocol.

### 2.2 Enzyme-linked immunosorbent assay (Elisa)

Elisa tests were performed using the dedicated Abnova kits for human serum albumin (Catalogue #KA0455) and Complement C3 (Catalogue #KA1020), following the manufacturer׳s protocols.

### 2.3 Statistical analysis

Receiver operating characteristics (ROC) curve analyses were performed to determine the best predictor of endometriosis between the tested variables. Statistical analysis was performed with the SPSS package (version 13.05, SPSS, Inc.).

## Conflict of interest

The authors (Pietro G. Signorile and Alfonso Baldi) declare that they have a patent application (WO 2013/171655) related to the themes of the article.

## Footnotes

Appendix A Supplementary data associated with this article can be found in the online version at doi:10.1016/j.dib.2015.10.047.
