# Supplementary material for: Supporting evidences for potential biomarkers of endometriosis detected in peripheral blood
Source: Data Brief. 2015 Nov 17;5:971–4. doi: 10.1016/j.dib.2015.10.047 (PMC4683550; doi:10.1016/j.dib.2015.10.047)
Supplement: Supplementary file 1 — Supplementary material [file mmc1.doc]

The authors (Pietro G. Signorile and Alfonso Baldi) declare that they have a patent application (WO 2013/171655) related to the themes of the article.
